# First case report of M1 macrophage polarization in an untreated symptomatic patient with toxoplasmosis

**DOI:** 10.1186/s12879-018-3048-2

**Published:** 2018-03-27

**Authors:** Graziano De Luca, Chiara Di Lisio, Giuseppe Lattanzio, Tommaso D’Antuono, Marcella Liberatore, Francesca Bianca Aiello

**Affiliations:** 1 Center for Excellence on Aging and Translational Medicine, University G. d’Annunzio, Chieti-Pescara, via dei Vestini, 66100 Chieti, Italy; 2 Department of Medicine ad Aging Sciences, University G. d’Annunzio, Chieti-Pescara, via dei Vestini, 66100 Chieti, Italy; 3 Department of Oncology, ASL2, SS Annunziata Hospital, via dei Vestini, 66100 Chieti, Italy

**Keywords:** Toxoplasma gondii, Macrophage polarization, T helper 1 immune response, Immunodeficiency

## Abstract

**Background:**

In immunocompetent patients, acute toxoplasmosis is usually asymptomatic. We identified M1 macrophages in a case of symptomatic acute *Toxoplasma gondii* infection that resolved without treatment. M1 macrophages have been demonstrated in animal models of toxoplasmosis, but not in humans.

**Case presentation:**

A 63-year-old woman presented with laterocervical and axillary bilateral lymphadenopathy. Her anamnesis defined an episode of high fever and prolonged asthenia 4 months previously, which suggested an infectious disease. Following laboratory, radiological, and pathological analyses, she was diagnosed with toxoplasmosis. Immunohistochemical analyses were performed on lymph node sections. More than 50% of the macrophages in the lymph node microgranulomas were M1 macrophages, defined by CD68^+^/p-Stat1^+^ staining, and the presence of T helper 1 lymphocytes indicated an immune response known to induce M1 macrophage polarization. Activated endothelial cells were found only in inflamed areas. No therapy was administered before or after diagnosis, and the lymphadenopathy resolved after a follow-up of 5 months.

**Conclusions:**

This is the first report to demonstrate the presence of M1 macrophages in human toxoplasmosis. Our findings contribute to the understanding of the pathogenesis of toxoplasmosis, and encourage further studies on the role of macrophage polarization in human toxoplasmosis.

## Background

Toxoplasmosis is a parasitic infection that can be life threatening in immunodeficient patients and when it is transmitted congenitally. In immunocompetent patients, the acute infection is mostly asymptomatic, and is clinically evident in only 10% of cases, presenting as a flu-like illness associated with lymphadenopathy. It is frequently benign and patients usually recover, although, chorioretinitis, hepatitis, myocarditis, or encephalitis can sometimes develop. In other cases, the acute infection can result in chronic persistence of cysts within the tissues of the hosts, for which an effective immune response is important [1, 2].

Macrophage polarization has different effects that influence the progression of inflammatory responses. M1 macrophages are activated by Toll receptor ligands and cytokines produced by T helper 1 lymphocytes, and they sustain inflammation [1]. M2 macrophages are activated by various cytokines, including IL-4 produced by T helper II lymphocytes, and they show a wide spectrum of functions, including angiogenesis, immune suppression, and tissue remodeling and repair [1, 3]. In vitro studies have suggested that a subset of M2 macrophages predominates during inflammation repair, and that a switch from one functional phenotype to another is possible in response to microenvironmental signals [1, 3].

Most studies that have focused on macrophage polarization have been performed in vitro or in animal models [1, 3], and thus the characterization of macrophages in human diseases is an important issue. M1 macrophages have been demonstrated in experimental models of *Toxoplasma gondii (T. gondii)* infection, although not in humans. Interferon gamma (IFN-γ) produced by T lymphocytes is the major mediator of resistance against *T. gondii* [4]. Activation of the transcription factor signal transducer and activator of transcription 1 (Stat1) is essential to mediate the antimicrobial effects of IFN-γ, particularly in the immune response against *T. gondii* [2]. Stat1-deficient mice are indeed highly susceptible to this disease [2]. In macrophages, IFN-γ induces Stat1 phosphorylation, dimerization and translocation to the nucleus, where it leads to the transcription of genes encoding proteins that are essential for the response to intracellular parasites (inducible nitric oxide synthase, also called iNOS, immunity-related GTPase and others) [2]. Combined immunoreactivity for nuclear phospho-Stat1 (p-Stat1) and the membrane macrophage CD68 marker has been shown to identify M1 macrophages in human tissues [5]. The CD68 antigen, which is specifically recognized by the PG-M1 monoclonal antibody, is considered the most specific marker available for all human macrophages [6, 7], thus a double CD68/p-Stat1 positivity clearly allows an accurate detection of the M1 macrophage subset. Using this method we identified M1 macrophages in a human *T. gondii* infection. Moreover, we observed CD31-positive endothelial cells with nuclear p-Stat1 immunoreactivity in areas with mononuclear cell infiltration, which suggests inflammation-induced endothelial cell activation.

## Case presentation

**Fig. 1:**
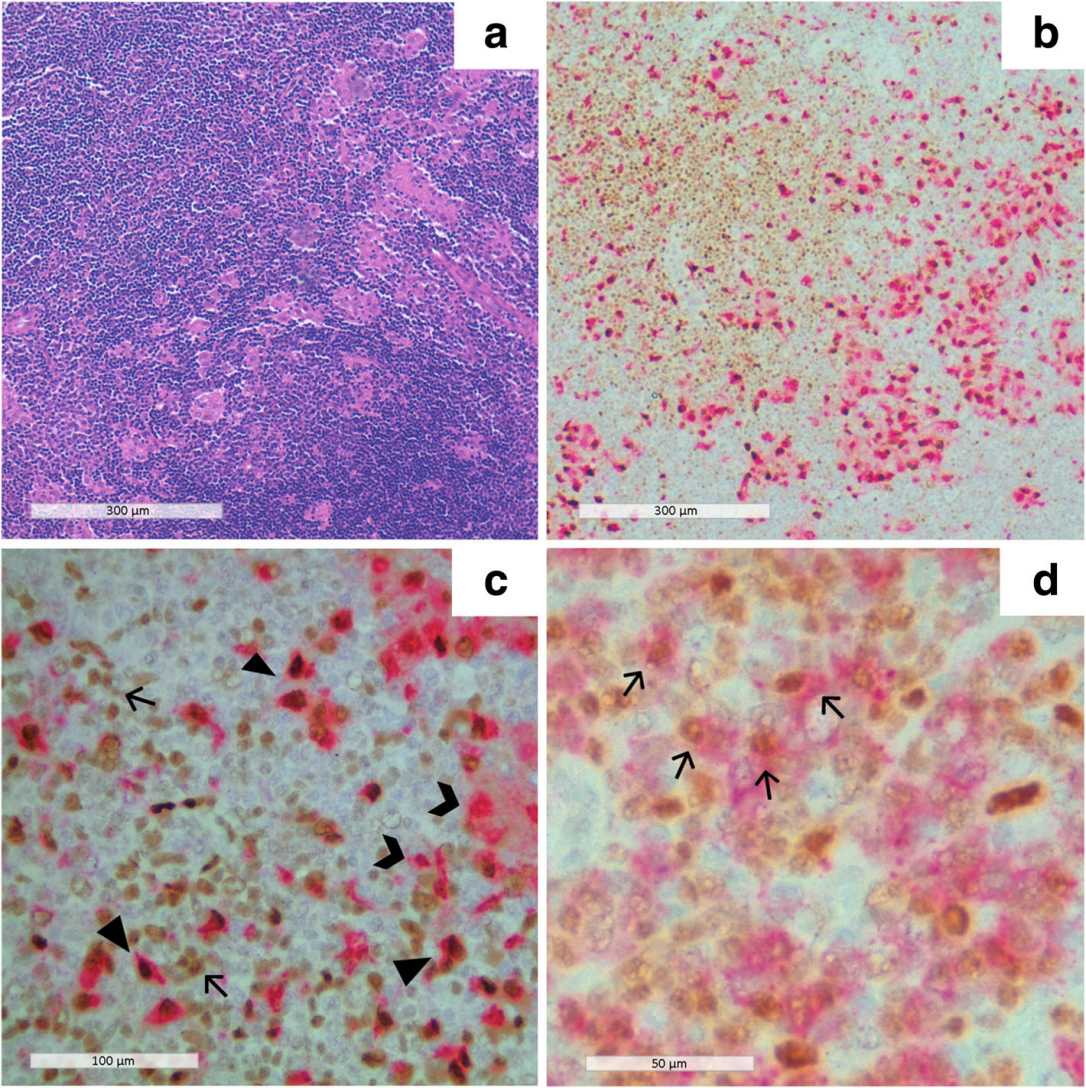
Morphological and immunohistochemical evaluation of inflammatory cells in axillary lymph nodes. **a** hematoxylin-eosin: scattered microgranulomas; **b** CD68 /pStat1 immunoreactivity: CD68+, pStat1+ and CD68/pStat1+ cells (CD68: red color, pStat1: brown color); **c** CD68/pStat1 double immunoreactivity: triangles, CD68 single immunoreactivity: guillemets, pStat1+ single immunoreactivity: arrows (CD68: red color, pStat1: brown color); **d** CD8/pStat1 immunoreactivity, arrows indicate /pStat1+ cells (CD8: red color, pStat1: brown color)

A 63-year-old nulliparous woman underwent mammary and axillary echography for breast cancer screening in January 2016. No lesions were detected in the mammary glands. However, a bilateral axillary lymphadenopathy was observed. Physical examination showed bilateral tumefaction of both axillary and laterocervical nodes. In February 2016 she underwent hematological counseling to exclude lymphoproliferative diseases. Her medical history revealed an episode of high fever that had lasted 1 week and a prolonged asthenia. This had occurred at the end of August 2015**,** and suggested an infectious disease. Her blood count showed lymphocytosis (3340 cells/mm^3^, 43%). Cytomegalovirus, Epstein Barr virus, human immunodeficiency virus, and human herpes virus 1/2 serological tests were negative. The anti-*T. gondii* IgM index was 0.80, slightly positive (> 0.60), and the IgG titer was 1448 UI/ml, considerably high, pointing to an acute *T. gondii* infection [8]. Computed tomography scan and, after a month, a surgical biopsy were performed which excluded lymphoid or epithelial tumors. Bilateral hilar and mediastinal lymphadenopathy were observed. Histological evaluation of the axillary lymph-nodes showed alterations of the normal architecture with marked follicular hyperplasia and many micro-granulomas composed of epithelioid histiocytes with abundant pale cytoplasm, encroaching on the mantle zone and germinal centers (Fig. 1a), and monocytoid B-cell hyperplasia enlarging the nodal sinuses. The triad of follicular hyperplasia, micro-granulomas, and monocytoid B-cell hyperplasia is considered highly specific for *T. gondii* infection [9]. Clinical, serological and histopathological findings thus revealed the late phase of an acute *T. gondii* infection. No therapy was administered before or after biopsy. The lymphadenopathy resolved after a follow-up of 5 months, and thus, the infection was self-limited. Immunohistochemistry was performed on lymph node sections (2-3 sections/staining). Murine anti-CD68 -CD3, -CD4, -CD8, -CD20, and -CD31 monoclonal antibodies (clones PG-M1, F7.2.38, 4B12, C8/144, L26 and JC70A, respectively) were used alone or in combination with a rabbit-anti-p-Stat1 monoclonal antibody (clone 58D6). Murine anti-iNOS monoclonal antibody (clone 4E5) and rabbit anti-IFN-γ polyclonal antibody were used alone to detect iNOS and IFN-γ immunoreactivity. For single red color staining after antigen retrieval (pH 6 or pH 9 as appropriate) sections were incubated with the primary antibody for 1 h at room temperature and then with a biotinylated rabbit anti-mouse secondary antibody (30 min) and streptavidin alkaline phosphatase (30 min). Alkaline phosphatase was visualized by incubation with Vulcan Fast Red (10 min). The staining was performed using the automated Omnis immunostainer (Dako, Glostrup, Denmark). For single brown color staining, after antigen retrieval sections were incubated with the primary antibody for 1 h, or overnight at room temperature, and then stained by the Envision technique (Dako). The staining was performed using the automated Autostainer Link 48 immunostainer (Dako) The single red and the single brown color stainings were performed sequentially for double staining. Cells were counted in 10 randomly selected high power fields (HPFs), with, numbers per HPF expressed as mean ± standard deviation.

**Fig. 2:**
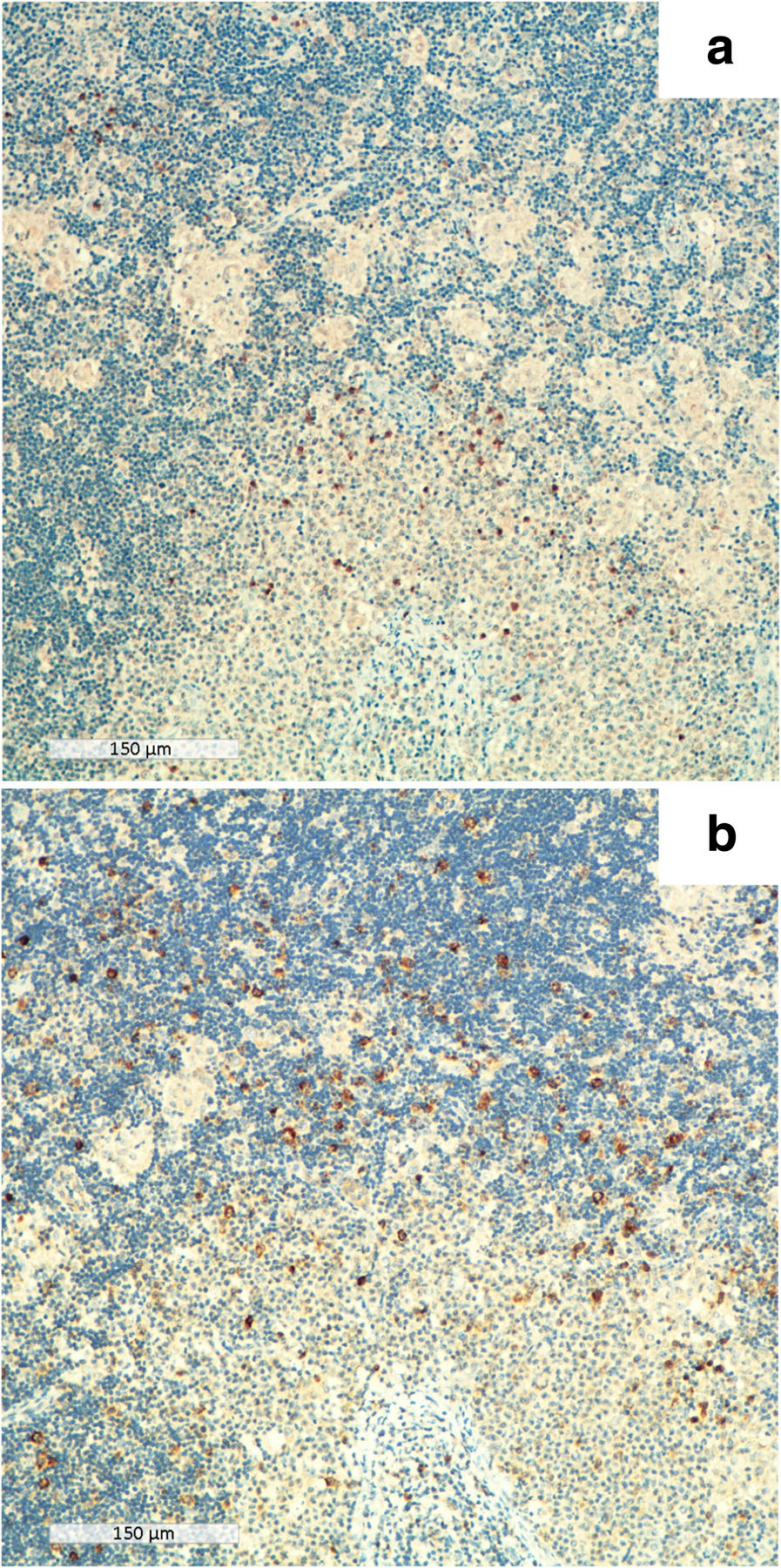
Immunohistochemical evaluation of IFN-γ and iNOS immunoreactive cells in axillary lymph nodes. **a** Scattered IFN-γ positive mononuclear cells between microgranulomas and in germinal centers; **b** Numerous iNOS positive mononuclear cells between microgranulomas and in germinal centers

To investigate whether M1 macrophages were involved in the inflammatory reaction lymph node sections were double stained with antibodies recognizing nuclear p-Stat1 and the membrane macrophage marker CD68 (Fig. 1b, c). In microgranulomas the mean number of CD68^+^ macrophages was 61.7 ± 24.3 per HPF. The mean numbers of CD68^+^ pStat1^+^ and CD68^+^ p-Stat1^−^ macrophages were 37.5 ± 22 and 28.2 ± 13 per HPF, respectively. The mean numbers of CD3-, CD4- and CD8-positive T-lymphocytes were 308.7 ± 139.5; 269.7 ± 130.9 and 120.3 ± 51.6 per HPF, respectively. Among the microgranulomas, a small number of p-Stat1^+^/CD68^−^ mononuclear cells were present (Fig. 1b) (48.1 ± 26 per HPF). CD3/p-Stat1 double staining confirmed the presence of p-Stat1^+^ T lymphocytes in these areas (13.5 ± 5.3 per HPF). A subset of the p-Stat1^+^ cells were CD8^+^ (6.8 ± 2.9 per HPF) (Fig. 1d). We did not assess the number of p-Stat1^+^/CD4^+^ cells because the intensity of the membrane positivity was low when the anti-CD4 antibody was used in double staining. CD20^+^ B lymphocytes were p-Stat1 negative (data not shown). Scattered IFN-γ^+^ mononuclear cells were observed between microgranulomas and in germinal centers (Fig. 2a). In these areas iNOS^+^ mononuclear cells were present (36.4 ± 14.9 per HPF) (Fig. 2b).

**Fig. 3:**
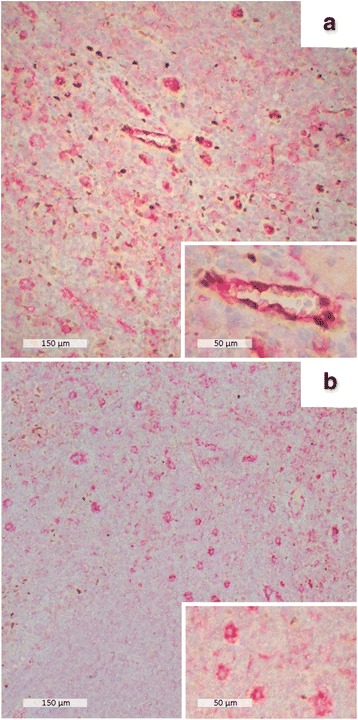
Immunohistochemical evaluation of endothelial cells in axillary lymph nodes. **a** endothelial cell CD31/pStat1 immunoreactivity in inflamed areas (CD68: red color, pStat1: brown color); **b** endothelial cell CD31/pStat1 immunoreactivity in areas without mononuclear cell infiltration. Insets in panel **a** and **b** show CD31/pStat1 immunoreactivity of endothelial cell at higher magnification

CD31^+^ endothelial cells showed a marked nuclear p-Stat1 immunoreactivity only in areas where p-Stat1^+^ mononuclear cells were present (Fig. 3a), whereas, in the absence of p-Stat1^+^ infiltrate, they were negative or very weakly positive (Fig. 3b).

## Discussion and conclusions

We found that in a human *T. gondii* infection more than 50% of the macrophages in microgranulomas were polarized as M1 macrophages during the IgM seroreversion/IgG seroconversion, which corresponds to the late phase of the inflammatory response. In this phase, a small percentage of T-lymphocytes were p-Stat1 immunoreactive. In addition, IFN-γ and iNOS immunoreactive cells showed a similar localization. Our observations suggest that in the acute phase of the anti-*T. gondii* immune response T helper 1 lymphocytes produced IFN-γ stimulating the activation of the p-Stat1 driven antimicrobial pathways in macrophages.

In mice, *T. gondii* replication in brain endothelial cells precedes invasion of the central nervous system [10]. Importantly, in vitro data have shown that cytokine-activated murine endothelium contributes to the clearance of *T. gondii* through recruitment of CD8^+^ lymphocytes [11]. We observed human endothelial cells that expressed p-Stat1 in inflamed areas, where pStat-1^+^ mononuclear cells were present. It is tempting to speculate that human activated endothelial cells contribute to the anti-*T. gondii* immune response. In the present patient the infection self-resolved, which suggested that the M1 response led to healing and might have a major role in promoting an effective response. Recent results in animal models indicate that M2 macrophage polarization in *T. gondii* infection may be deleterious and be associated with chronic persistence [12].

Although our findings concern a single patient and cannot be generalized, they encourage studies about the role of macrophage polarization in toxoplasma chronic persistence and reactivation in human subjects.

Primary *T. gondii* infection in pregnancy is treated with spiramycin alone or in combination with other drugs, and the pooled rate of vertical transmission in treated patients after therapy is about 10% [13]. Anti-parasitic treatment suppresses the production of specific IgM and IgG in mice [14] and decreases the production of IgG in pregnant women [15]. Whether anti-parasitic therapy affects macrophage polarization remains to be investigated. To date, all studies concerning macrophage polarization in toxoplasmosis have been performed in experimental animal models. We identified for the first time a large number of M1 macrophages in a symptomatic case of human toxoplasmosis that spontaneously resolved.

## Abbreviations

IFN-γ: Interferon gamma
iNOS: Inducible nitric oxide synthase
pStat1: Phospho-signal transducer and activator of transcription 1
Stat1: Signal transducer and activator of transcription 1
*T. gondii*: *Toxoplasma gondii*
